# Experimental Investigation and Thermodynamic Verification for the Phase Relation around the ε-Mg_23_ (Al, Zn)_30_ Intermetallic Compound in the Mg-Zn-Al System

**DOI:** 10.3390/ma14226892

**Published:** 2021-11-15

**Authors:** Yan Zheng, Jiaxing Sun, Kaiming Cheng, Jin Wang, Chengwei Zhan, Jingrui Zhao, Xitao Wang, Shouqiu Tang, Jixue Zhou, Lijun Zhang, Yong Du

**Affiliations:** 1 Shandong Provincial Key Laboratory of High Strength Lightweight Metallic Materials, Advanced Materials Institute, Qilu University of Technology (Shandong Academy of Sciences), Jinan 250014, China; zhengyan08212013@163.com (Y.Z.); jiaxingsung@foxmail.com (J.S.); wangjin@sdas.org (J.W.); chengwei.zhan@sdas.org (C.Z.); xtwang@ustb.edu.cn (X.W.); tangshq@sdas.org (S.T.); yong-du@csu.edu.cn (Y.D.); 2 Laboratory of Materials Phase Equilibria and New Materials Design, School of Materials Science and Engineering, University of Science and Technology Beijing, Beijing 100083, China; 3 School of Materials Science and Engineering, Shandong Jianzhu University, Jinan 250101, China; jingr_zhao@126.com; 4 State Key Laboratory of Powder Metallurgy, Central South University, Changsha 410083, China; lijun.zhang@csu.edu.cn

**Keywords:** Mg-Zn-Al alloy, ε phase, phase equilibrium, calculation of phase diagram (CALPHAD), diffusion

## Abstract

The appearance of the ε phase during the welding process can severely weaken the welding strength of dissimilar metals of Mg-Zn-Al alloy systems. An understanding of the accurate phase diagram, especially the equilibrium phase relation around the ε phase, is thus of particular importance. However, the phase interrelation near the *ε*-Mg_23_(Al, Zn)_30_ phase has not yet been fully studied. In this work, the local phase diagrams of the ε phase and its surrounding phases in the Mg-Zn-Al system are systematically determined by experimental investigation and thermodynamic verification. Five Mg-Zn-Al alloys and one diffusion couple were fabricated and analyzed to get accurate phase constituents and relationships adjacent to ε phase. The current experimental data obtained from Scanning Electron Microscope (SEM), X-ray diffraction (XRD), Differential Scanning Calorimetry (DSC), and Electron Probe Micro Analysis (EPMA) were further compared with the thermodynamically computed phase relations around ε phase for verification, showing good agreements. Several important conclusions are drawn based on current experimental work, which can provide supporting information for the follow-up studies on ε phase in the Mg-Zn-Al alloy systems.

## 1. Introduction

Due to their relatively low density, good specific stiffness, specific strength and electromagnetic shielding, biocompatibility, recyclability, large hydrogen storage capacity, and high theoretical specific capacity for battery, magnesium alloys have attracted more and more attention for their application in the automotive, aerospace, biomedical, and energy industries [1,2,3,4,5,6]. For decades, efforts have been made to improve the mechanical properties, creep resistance and electrochemical stability of magnesium alloys, in order to broaden their practical applications. One of the most common methods is to design the alloy composition so as to modify the microstructure [7], and controlling over the formation of various intermetallic compounds (IMCs) is essential. In Mg-Zn-Al alloy systems, the existence of binary (*γ*-Mg_17_Al_12_, *ε*-Mg_23_Al_30_, and *β*-Al_3_Mg_2_) and ternary (*φ*-Mg_5_Al_2_Zn_2_ and *τ*-Mg_32_ (Al, Zn)_49_) intermetallic compounds can be manipulated to adjust the alloy performance. For instance, the presence or absence of Mg_17_Al_12_ precipitates has a great influence on the grain structure upon hot deformation, thereby affecting the strength of the deformed magnesium alloys [8]. The discontinuous precipitation of coarse γ-Mg_17_Al_12_ phase in AZ alloys causes softening of the grain boundary at high temperatures, reducing the strength and creep resistance, thus limiting the service temperature to below 120 °C [9,10,11,12]. It has been found that the high temperature creep resistance of ZA series alloy is much better than that of AZ91 [13]. The unfavorable Mg_17_Al_12_ phase can be replaced by the thermally stable Mg-Zn-Al ternary phases, such as the *τ*-Mg_32_(Al, Zn)_49_ phase and *φ*-Mg_5_Al_2_Zn_2_ phase, at high temperatures, making the creep behavior of ZA magnesium alloys with high Zn content better than that of AZ alloys [14,15]. Shi et al. [16] reported that the age hardening response of Al in Mg-6Zn-5Al alloys can be further improved due to the existence of τ-Mg_32_(Al, Zn)_49_ phase. Other intermetallic compounds, such as ε-Mg_23_Al_30_, can be detected in the welding process. For example, the ε phase was detected in the central region of the friction stir welding of AZ31 and 6061 aluminum alloys [17,18]. The formation of Mg_23_Al_30_ phase should be avoided, since it is the main weakness in the welding strength of Mg-Al dissimilar metals, as stated by Sun et al. [19]. The formation of *ε*-Mg_23_(Al, Zn)_30_ phase, where Zn partially occupies the sublattice of Al in the binary Mg_23_Al_30_ phase, was also detected by Wang et al. [20] when studying the welding coating of Mg-Al*_x_*Zn_1-*x*_ alloys using the diffusion couple technique. An understanding of the accurate phase diagram, especially the equilibrium phase relation among different intermetallic compounds for different alloy composition, is of particular importance during the alloy design for the Mg-Zn-Al system.

There are several experimental studies on the phase diagram of Mg-Zn-Al alloys. Eger et al. [21] presented the first systematic investigation on the liquidus surface. Bergman et al. [22,23] and Clark et al. [24,25] then studied the ternary intermetallic compounds of τ phase and φ phase, respectively. Later, the first summarizing review was presented by Willey et al. [26], based on which Liang et al. [27] and Liang et al. [28] presented detailed thermodynamic models of the Mg-Zn-Al system, respectively. More recent experimental studies include the isothermal section of the Mg-rich corner in the Mg-Zn-Al system determined at 300 °C, 320 °C, and 335 °C by Ren et al. [29,30,31], and the Mg-rich phase equilibria and solidification behaviors studied by Ohno et al. [32]. Although there is a large volume of experimental data of the Mg-Zn-Al phase diagram, the phase interrelation near the *ε*-Mg_23_ (Al, Zn)_30_ phase has not yet been fully studied. In this work, we present an experimental investigation and thermodynamic calculation of the Mg-Zn-Al system, with a particular focus on the *ε*-Mg_23_ (Al, Zn)_30_ phase and its equilibrium phase relations. Alloys with different compositions and diffusion couples were fabricated and analyzed by Scanning Electron Microscope (SEM), X-ray diffraction (XRD), Differential Scanning Calorimetry (DSC), and Electron Probe Micro Analysis (EPMA). The current experimental results, together with the data from previous research, were then compared with the available thermodynamic description of the Mg-Zn-Al system for further discussion.

## 2. Experimental Procedure

In this study, five alloys with different compositions were prepared from high-purity Mg (99.9%), Al (99.99%), and Zn (99.99%), which were melted in an argon atmosphere by resistance furnace. The nominal compositions of the samples were selected according to the isothermal section at 350 °C, which was calculated based on the previous thermodynamic description [27] containing the *ε*-Mg_23_ (Al, Zn)_30_ phase, including the ε, ε + γ, ε + β, ε + γ + τ, and ε + β + τ phase regions, as shown in Table 1. During alloying, the resistance furnace was first heated up to 710 °C, and the Al blocks were placed in a stainless-steel crucible in the controlled atmosphere furnace before the addition of Mg and Zn. The samples were then heated up to 720 °C and held for 25 min before being cooled down to 690 °C before casting. The heating process was carefully controlled to avoid massive volatilization. The melts poured in graphite crucibles for casting were then annealed at 350 °C for 305 h in an argon atmosphere to reach homogenization.

Two end members, i.e., MgZn_2_ and Mg_17_Al_12_, were selected in this work to form a diffusion couple, in order to observe the diffusion path across *ε*-Mg_23_ (Al, Zn)_30_ phase. The end-member alloys of intermetallic compounds were fabricated according to the stoichiometric ratio using an identical processing route to that detailed in our previous work [14]. The alloys were annealed at 450 °C for 12 h in an argon atmosphere, and then cut into 8 mm × 5 mm × 3 mm blocks before being polished in ethanol. The treated surface was bonded with a tantalum clamp before it was sealed in the vacuum quartz tube to avoid oxidation during the diffusion annealing process. The packaged diffusion couple was annealed in a 410 °C furnace for 4 h to form intermetallic compound layers with a temperature error within ± 1 °C.

The annealed alloy samples were investigated by XRD (Rigaku D-Max/2550VB+, Rigaku, Japan) and SEM (Zeiss EVO M10, Zeiss, Germany) for phase identification. The compositions of the annealed samples and the phase constitutions were determined by EPMA (JXA-8230, JEOL, Japan). These samples were further subjected to DSC analysis (NETZSCH STA449F3A, NETZSCH, Germany) to detect the phase transformation temperature, where a heating rate of 10 K/min and a temperature range of 32 °C to 600 °C were applied. The annealed diffusion couples were unclamped from the Ta jigs and subjected to wire-cutting parallel to the diffusion direction. The cutting surfaces were metallographically prepared for analysis of the microstructure and diffusion path by SEM and EPMA.

## 3. Results and Discussion

### 3.1. Phase Equilibrium Containing ε Intermetallic Compound

**Figure 1:**
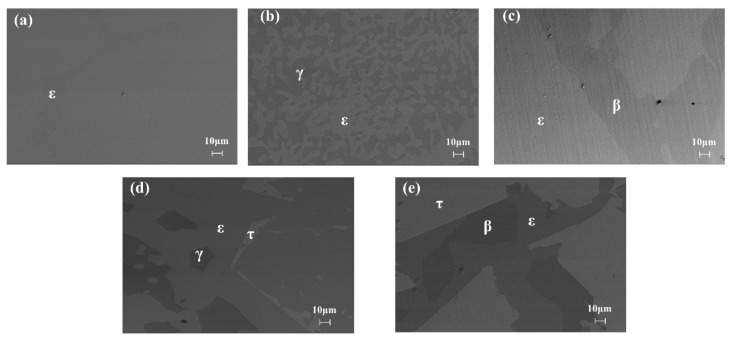
SEM images of samples annealed at 350 °C for 305 h: (**a**–**e**) represent samples 1, 2, 3, 4, and 5, respectively.

**Figure 2:**
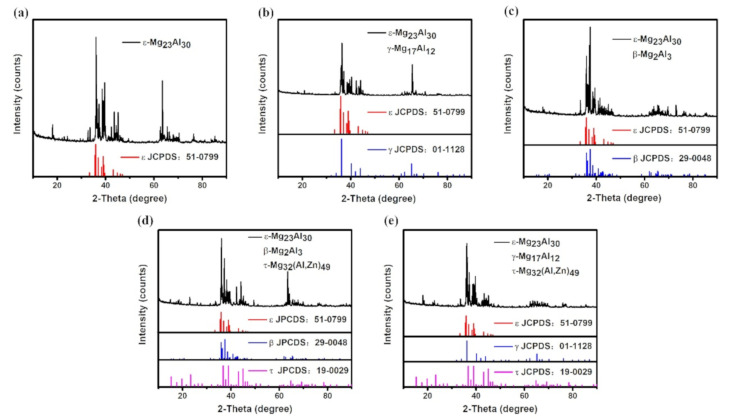
XRD patterns of samples annealed at 350 °C for 305 h: (**a**–**e**) represent samples 1, 2, 3, 4, and 5, respectively.

Figure 1 shows the SEM images of the five alloy samples after heat treatment, and the corresponding XRD patterns are summarized in Figure 2. Table 1 lists the composition of phases that appeared in each sample measured by EPMA. The phase constitutions of each alloy can be determined by analyzing all the SEM images, XRD patterns, and EPMA results. It can be seen from Figure 2a that sample 1 contained a single ε phase, which was consistent with the uniform microstructure of Figure 1a. The EPMA results in Table 1 indicate a solid dissolution of 3.9% Zn in the ε single phase. From Figure 1b, it can be seen that the light gray and dark gray phases distributed evenly in sample 2, which was selected to be within a two-phase region of ε and γ. According to the XRD and EPMA analysis, as shown in Figure 2b and Table 1, the two phases were determined as γ phase and ε phase, respectively. The content of Zn in ε phase was 4.05% when ε phase is in equilibrium with γ phase. In Figure 1c, it can be observed that sample 3 contains bright and dark phase regions with clear phase boundaries, which are ε and β phase according to the XRD patterns shown in Figure 2c. The ε phase is brighter than the β phase due to the higher content of Zn (3.27% in ε phase and 2.64% in β phase). The SEM image of Figure 1d shows a three-phase region with different colors of bright, light gray, and dark gray. Upon analysis of the EPMA result and the XRD pattern shown in Figure 2d, the bright, light gray, and dark gray phase regions corresponded to τ, ε, and γ phase, respectively. That is, sample 4 is a ternary alloy within the three-phase equilibrium of ε + γ + τ, showing a large solid solubility of 7.7% Zn in ε phase. Figure 1e exhibits an obvious three-phase region in sample 5. According to Figure 2e and Table 1, the light, dark, and light gray phase regions correspond to τ, β, and ε phase, respectively, and the solid solubility of Zn in ε phase is determined as 7.03%.

**Figure 3:**
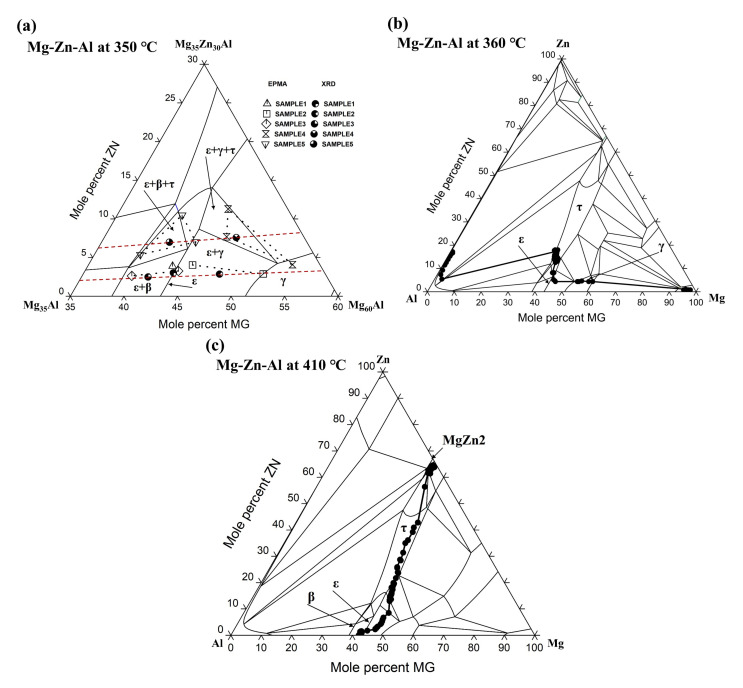
Isothermal section of the Mg-Zn-Al system (**a**) close to the ε phase calculated at 350 °C along with the data from XRD and EPMA; (**b**) calculated at 360 °C along with the diffusion profile of Al-Mg20Zn from literature; (**c**) calculated at 410 °C along with the diffusion profile of MgZn_2_-Al_3_Mg_2_ diffusion couple.

The experimental data points obtained by analyzing the XRD patterns and EPMA results are shown in Figure 3a, along with the isothermal section calculated at 350 °C. The nominal compositions of samples 1–5 are within the corresponding phase regions of ε, ε + γ, ε + β, ε + γ + τ, and ε + β + τ, which confirms that the phase constitutions of the currently prepared samples are located in the desired phase region of the Mg-Zn-Al alloy system. Tie lines for the phase regions of each alloy sample are also plotted, showing reasonable agreements with the calculated isothermal section. There are two three-phase equilibrium regions directedly connected with ε phase, which can be seen from samples 4 and 5 in the phase regions of ε + γ + τ and ε + β + τ. The maximum solid solubility of Zn in ε phase was determined to be 7.7% at 350 °C.

### 3.2. Vertical Sections around ε Intermetallic Compound

**Figure 4:**
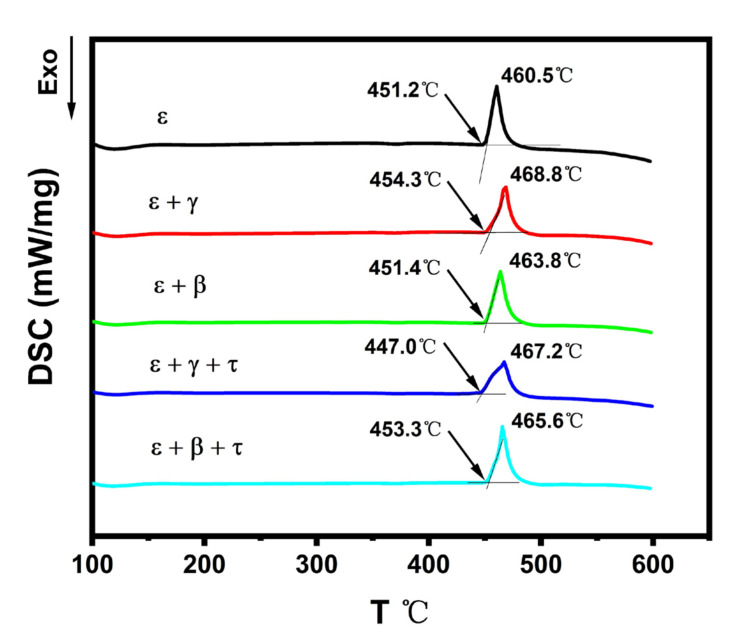
Current DSC curves of samples 1–5 with a heating rate of 10 K min^−1^.

**Figure 5:**
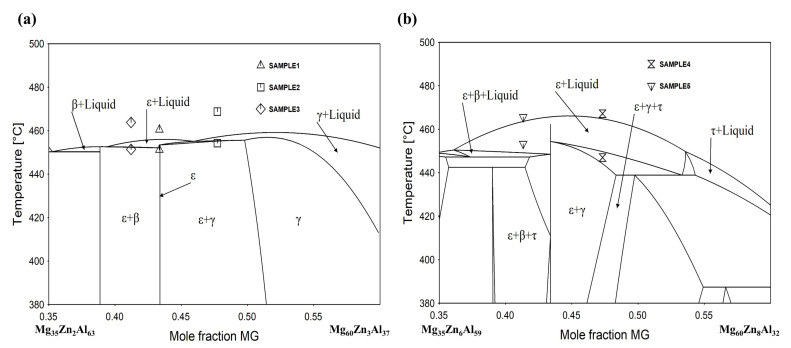
Calculated vertical section of the Mg-Zn-Al system (**a**) along with the DSC data of samples 1–3 and (**b**) along with the DSC data of samples 4 and 5.

In order to study the extension of ε phase relation in the direction of temperature, the phase transformation temperatures in samples 1–5 were then measured by DSC. The experimental heating curves of all five samples determined by DSC are shown in Figure 4. The original DSC data are presented in the Supplementary Materials. The phase transformation temperatures were obtained by acquiring the intersection point of the tangent lines at the initial onset temperature, while the peak values were determined to be the liquidus temperatures. These data are also shown in Figure 5, where the vertical sections across samples 1–3 and 4–5 are calculated respectively for comparison. These two vertical sections are marked using red dashed lines in Figure 3a.

It can be seen that the invariant equilibria temperature and liquidus temperature for sample 1, i.e., single ε phase, are 451.2 °C and 460.5 °C. These two temperatures correspond to the calculated vertical section in Figure 5a, where the phase should first enter into a two-phase region of liquid + ε at 451 °C before being completely melted at 461 °C. For samples 2 and 3, the temperature for eutectic reactions L = ε + γ and L = ε + β were measured to be 454.3 °C and 451.4 °C, showing a good agreement with the calculated vertical section in Figure 5a. However, the measured melting temperatures for samples 1–3 were about 10 K higher than the calculation results. The deviation can be attributed to the relatively fast heating rate of 10 K/min during the DSC analysis, where signals of the liquidus overlapped with the invariant reaction temperature. In Figure 5b, the observed thermal signals at 447 °C and 453.3 °C correspond to the temperatures when samples 4 and 5 entering the ε + L two phase region, respectively. The measured melting temperatures of 467.2 °C and 465.6 °C are in good agreement with the calculation results. Based on the calculated vertical section of Figure 5, it can be further noted that the ε phase with the dissolved 3.9% Zn (sample 1) persisted up to 456 °C, and this temperature range extended to 465 °C when the 7.7% Zn (sample 4) dissolved in ε phase, indicating that Zn is a stabilizer for ε phase within its solid solubility.

### 3.3. Diffusion Path Related to ε Intermetallic Compound

In this study, the composition profiles of two diffusion couples, one from previous experimental work by Wang et al. [20] and the other from our experiment, were presented, along with the calculated isothermal section of the Mg-Zn-Al system at certain temperatures, to further verify the phase relations around ε intermetallic compounds.

Figure 3b shows the diffusion path of the diffusion couple Mg-Al20Zn determined at 360 °C in previous research [20], where the diffusion profile moves across the hcp-Mg, γ, τ, and ε single-phase region in sequence. According to [20], Zn was found in all three IMC layers of γ, τ, and ε, and its content increased discontinuously towards the Al-Zn substrate. The average composition of the thickest layer formed adjacent to the Al-Zn side of the diffusion couple was Al_45_Mg_40_Zn_15_, which is further verified by TEM characterization to be the τ-(Al, Zn)_49_Mg_32_ phase. The β-Al_3_Mg_2_ phase usually seen in Al-Mg binary systems was replaced by τ phase in this diffusion, indicating that high Zn content can be sufficient to change the diffusion path to fully suppress the formation of the undesirable β phase. The addition of Zn can significantly retard the thickening rate of the γ-Al_12_Mg_17_ phase, while it is weak at inhibiting the thickening rate of the overall IMC reaction layer in an Al-Mg diffusion couple. It can be further noted from the diffusion path that after entering the ε single-phase region from γ phase, the content of Zn increases clearly from 5% Zn to 8% Zn before entering the τ phase region, which is in good agreement with the current maximum solid solubility of 7.7% Zn determined form the alloy sample 4, as shown in Table 1.

**Figure 6:**
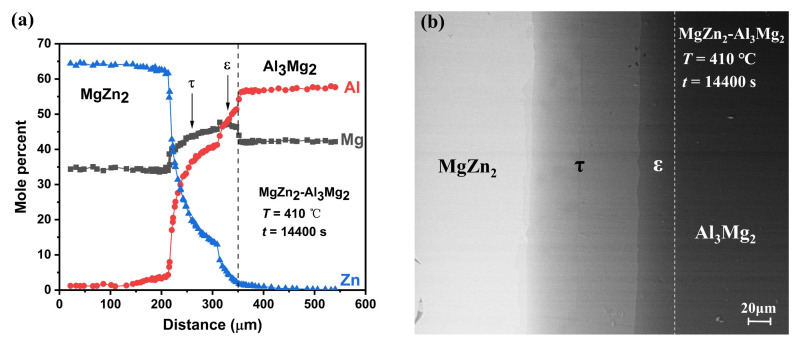
(**a**) EPMA data of MgZn_2_-Al_3_Mg_2_ diffusion couple annealed at 410 °C for 4 h. (**b**) SEM image of MgZn_2_-Al_3_Mg_2_ diffusion couple annealed at 410 °C for 4 h.

Figure 6 shows the EPMA and SEM results of the MgZn_2_-Al_3_Mg_2_ diffusion couples used in this study after annealing at 410 °C for 4 h, where the composition profile corresponding to the metallographic structure can be detected in detail. Figure 6a presents the EPMA results of a series of points selected on a line perpendicular to the phase interface along the diffusion direction, where the two interlayers of τ and ε phase can be detected. In Figure 6b, layers of the intermetallic compounds τ phase can be easily observed between MgZn_2_ and Al_3_Mg_2_, while ε phase is difficult to distinguish without noticing the composition jump between τ and Al_3_Mg_2_ phase, as shown in Figure 6a. Comparing the two figures, it can be seen that the thickness of τ phase is greater than that of ε phase, indicating a faster elemental diffusion within the τ phase. This situation agrees well with the experimental detection by Wang et al. [20]. It can be further noticed that the composition profiles within τ phase and ε phase exhibit clear nonlinear distributions, implying that the diffusivities of the elements are strongly composition-dependent.

The composition profile in Figure 6a is further plotted in the isothermal section of the Mg-Zn-Al system computed at 410 °C to detect the diffusion path, as shown in Figure 3c. It can be seen that ε phase lies between τ and Al_3_Mg_2_ phase, exhibiting a wide solid solubility range of Zn. The maximum homogeneity content of Zn in ε phase is 8.5% on the diffusion path, which is similar to the measured value of the current alloy sample 4 in Table 1. By comparing sample 4 with sample 1, the increment of Zn content from 3.9% to 7.7% leads to a clear decrease in Al content from 53.18% to 41.38%, indicating the Zn atoms dissolved in ε phase preferred to occupy the sites of the Al atoms. This result is consistent with the previous experimental findings by Wang et al. [20]. However, the diffusion path of the experimental data in this study deviates from the calculated single ε phase region to exhibit a higher Mg content, which was also detected in sample 4, where the Mg content ε phase (46.38%) is higher than that of sample 1 with a single ε phase (42.92%). As a result, there should be a homogenization range of Mg in the ε phase from 43.77 % (sample 5) to 46.38 % (sample 4) within the isothermal section, which may have been due to the Zn dissolution. Further modification of the thermodynamic description of Mg-Zn-Al is required to take these experimental data into consideration.

## 4. Conclusions

The phase diagram of the Mg-Zn-Al system focusing on the ε intermetallic compound and its surrounding phase relationships was obtained through experimental study and thermodynamic calculation. Some important conclusions are highlighted as follows.

- The existence of ε, ε + γ, ε + β, ε + γ + τ, and ε + β + τ phase regions in the isothermal section of the Mg-Zn-Al alloy system were confirmed by the analysis of the currently prepared samples. The maximum solid solubility of Zn in ε phase was determined as 7.7% at 350 °C.
- The melting temperature of ε phase with dissolved 3.9% Zn was 456 °C, which increased to 465 °C as the Zn content rose to 7.7%, indicating that Zn can improve the high temperature stability of ε phase within its solid solubility.
- Zn atoms dissolved in ε phase preferred to replace the sites of the Al atoms to decrease the total content of Al, and the homogenization range of Mg in the ε phase was from 43.77% to 46.38%. Further modification of the thermodynamic description of Mg-Zn-Al in this region is required.
- The MgZn_2_-Al_3_Mg_2_ diffusion couple at 410 °C in this study showed a thicker interlayer of τ phase than of ε phase, indicating a faster elemental diffusion within τ phase. Moreover, the nonlinear elemental distributions within τ and ε IMCs imply the composition dependence of elemental diffusivities.

## Figures and Tables

**Table 1:** The nominal compositions of current Mg-Zn-Al alloys and the phases constitutions and concentrations determined by XRD and EPMA.

Samples	Nominal Compositions (at.%)			Phases and Composition (at.%) at 350 °C			
	Mg	Zn	Al	Phase	Mg	Zn	Al
1	43.35	3.04	53.61	ε	42.92	3.90	53.18
2	47.73	2.87	49.40	ε	44.72	4.05	51.23
				γ	51.80	2.89	45.31
3	41.21	2.50	56.29	ε	43.72	3.27	53.01
				β	39.64	2.64	57.72
4	47.32	7.60	45.08	ε	46.38	7.70	45.93
				γ	54.04	4.09	41.87
				τ	45.03	11.28	43.69
5	41.33	7.00	51.68	ε	43.77	7.03	49.20
				β	39.29	5.39	55.32
				τ	41.06	10.50	48.44

## Data Availability

All data are available from the corresponding author on reasonable request.

## Supplementary Materials

The following are available online at https://www.mdpi.com/article/10.3390/ma14226892/s1, Figure S1: The DSC primary data of sample 1 (ε), Figure S2: The DSC primary data of sample 2 (ε + γ), Figure S3: The DSC primary data of sample 3 (ε + β), Figure S4: The DSC primary data of sample 4 (ε + γ + τ), Figure S5: The DSC primary data of sample 5 (ε + β + τ).
