# Supplementary material for: Experimental Investigation and Thermodynamic Verification for the Phase Relation around the ε-Mg23 (Al, Zn)30 Intermetallic Compound in the Mg-Zn-Al System
Source: Materials (Basel). 2021 Nov 15;14(22):6892. doi: 10.3390/ma14226892 (PMC8625525; doi:10.3390/ma14226892)
Supplement: Supplementary file 1 [file materials-14-06892-s001.zip › materials-1404998-supplementary.pdf]

Supplementary Materials

Experimental Investigation and Thermodynamic Verification for the Phase Relation Around the ε-Mg_{23} (Al, Zn)$_{30}$ Intermetallic Compound in the Mg-Zn-Al System

Yan Zheng [1], Jiaxing Sun [1,2], Kaiming Cheng [1,*], Jin Wang [1], Chengwei Zhan [1], Jingrui Zhao [3], Xitao Wang [1], Shouqiu Tang [1], Jixue Zhou [1,*], Lijun Zhang [4] and Yong Du [1,4]

[1] Shandong Provincial Key Laboratory of High Strength Lightweight Metallic Materials, Advanced Materials Institute, Qilu University of Technology (Shandong Academy of Sciences), Jinan 250014, China; zhengyan08212013@163.com (Y.Z.); jiaxingsung@foxmail.com (J.S.); wangjin@sdas.org (J.W.); chengwei.zhan@sdas.org (C.Z.); xtwang@ustb.edu.cn (X.W.); tangshq@sdas.org (S.T.); yong-du@csu.edu.cn (Y.D.)

[2] Laboratory of Materials Phase Equilibria and New Materials Design, School of Materials Science and Engineering, University of Science and Technology Beijing, Beijing 100083, China

[3] School of Materials Science and Engineering, Shandong Jianzhu University, Jinan 250101, China; jingr_zhao@126.com

[4] State Key Laboratory of Powder Metallurgy, Central South University, Changsha 410083, China; lijun.zhang@csu.edu.cn

* Correspondence: chengkm@sdas.org (K.C.); zhoujx@sdas.org (J.Z.)

Supplementary Figures

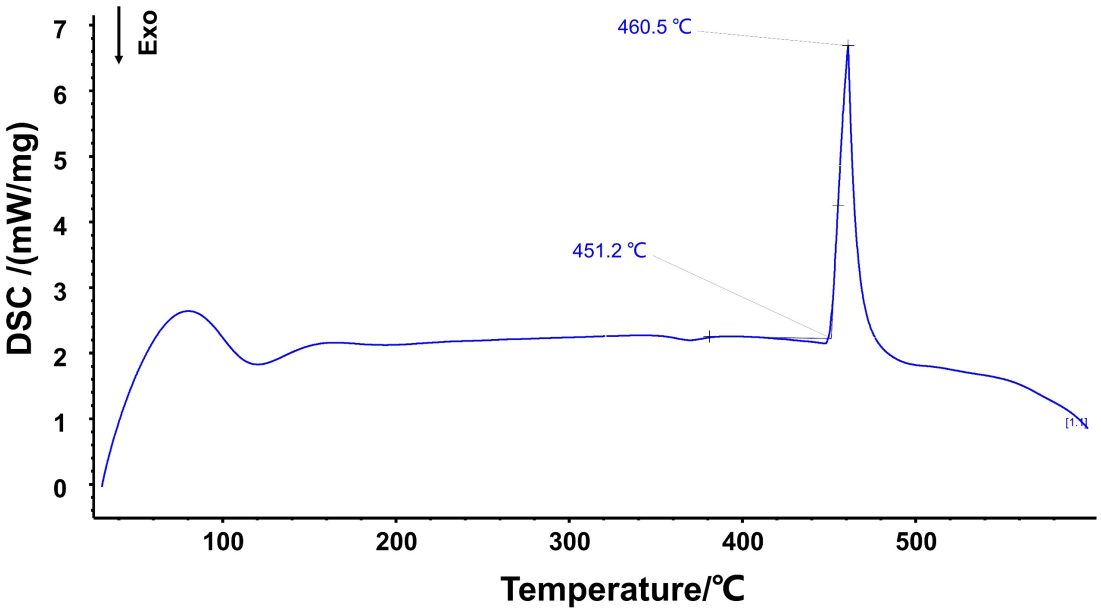

Figure S1. The DSC primary data of sample 1 (ε).

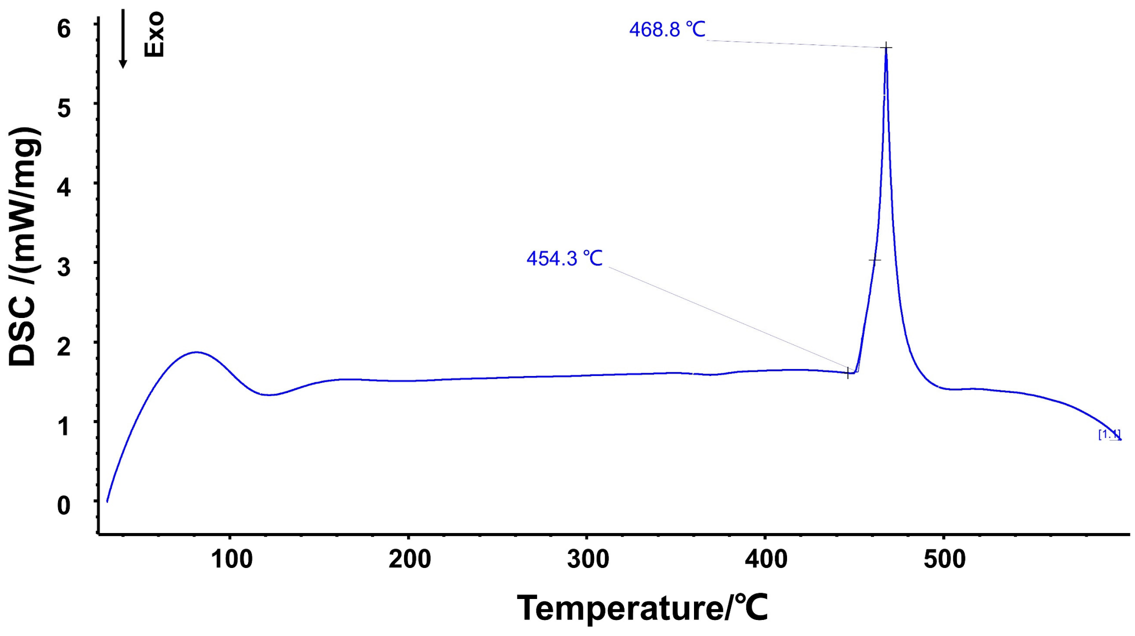

Figure S2. The DSC primary data of sample 2 (ε + γ).

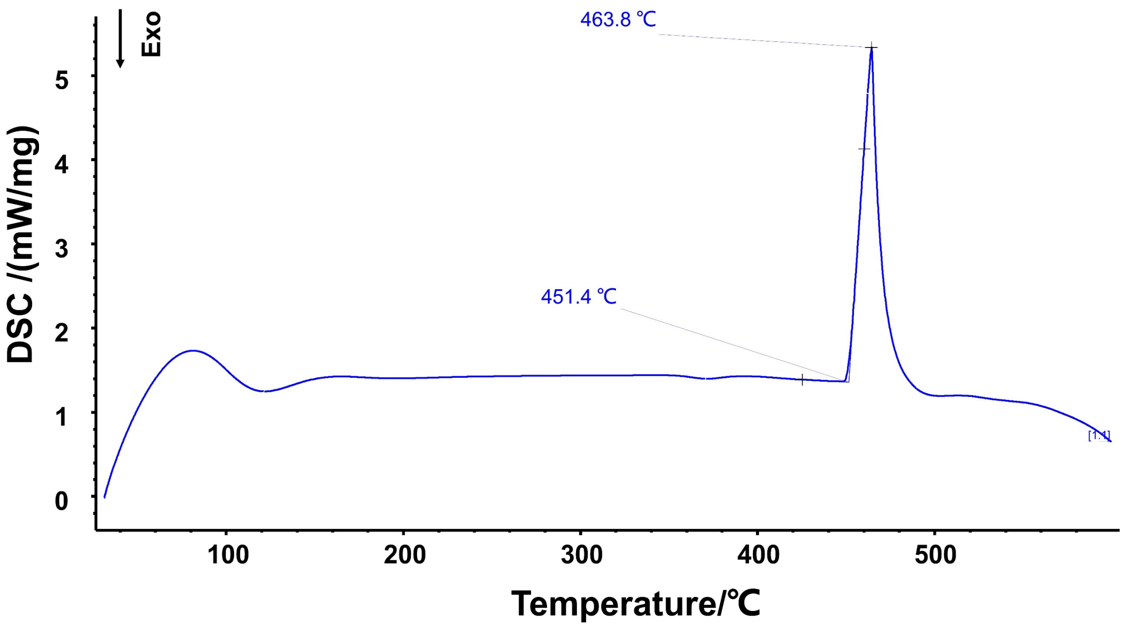

Figure S3. The DSC primary data of sample 3 (ε + β).

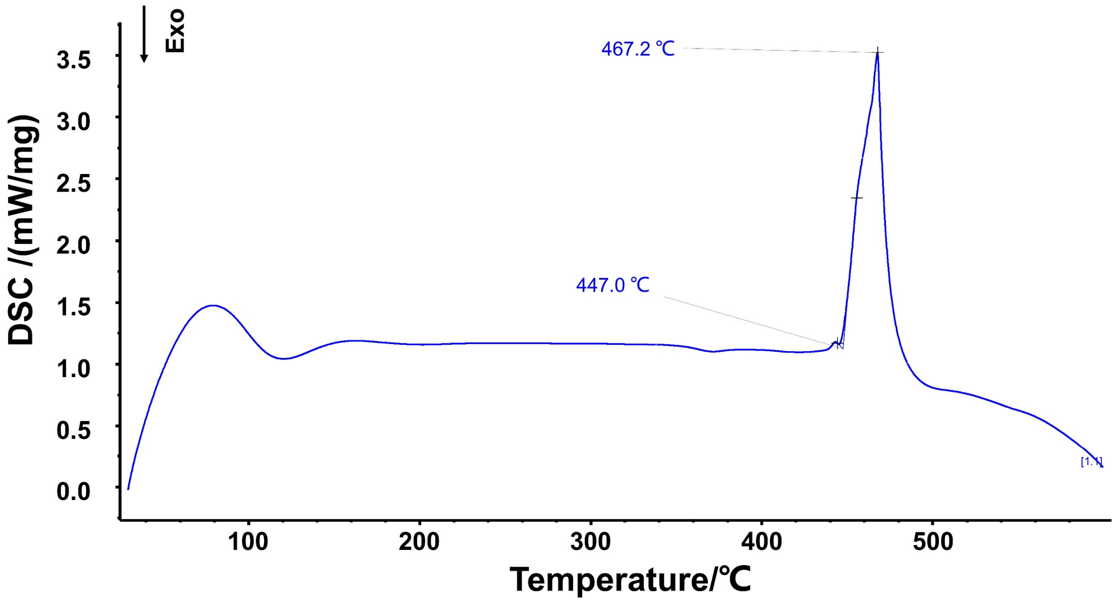

Figure S4. The DSC primary data of sample 4 (ε + γ + τ).

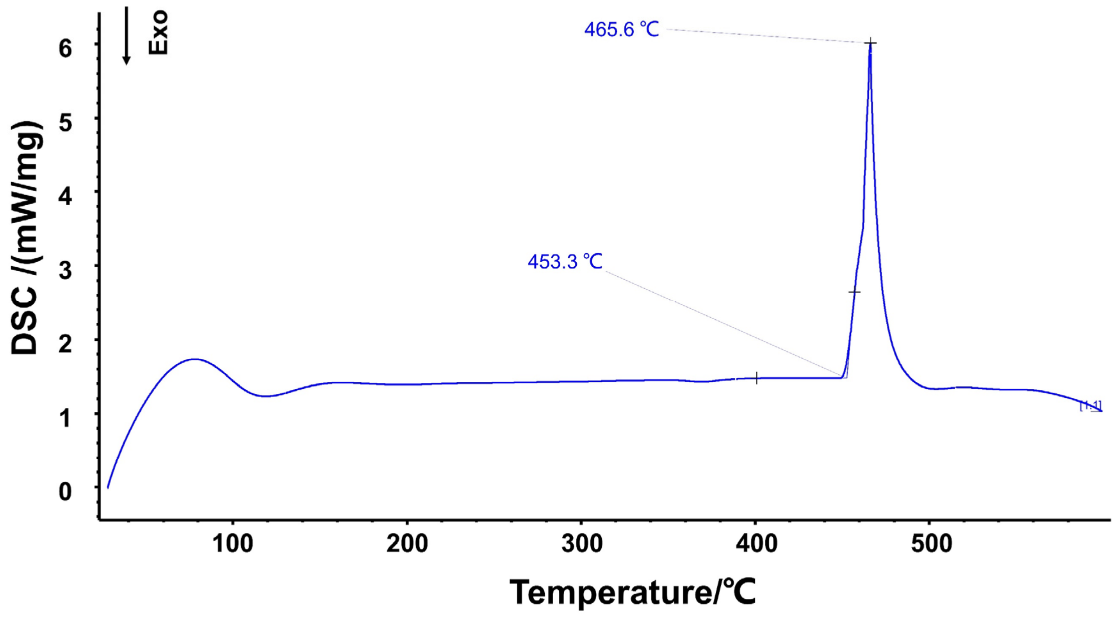

Figure S5. The DSC primary data of sample 5 (ε + β + τ).
